# Enhanced Delivery of Therapeutic siRNA into Glioblastoma Cells Using Dendrimer-Entrapped Gold Nanoparticles Conjugated with β-Cyclodextrin

**DOI:** 10.3390/nano8030131

**Published:** 2018-02-27

**Authors:** Jieru Qiu, Lingdan Kong, Xueyan Cao, Aijun Li, Ping Wei, Lu Wang, Serge Mignani, Anne-Marie Caminade, Jean-Pierre Majoral, Xiangyang Shi

**Affiliations:** 1 Department of Radiology, Shanghai Tenth People’s Hospital, Tongji University School of Medicine, Shanghai 200072, China; jieruq@outlook.com; 2 College of Chemistry, Chemical Engineering and Biotechnology, Donghua University, Shanghai 201620, China; lingdankong@hotmail.com (L.K.); caoxy_116@dhu.edu.cn (X.C.); andrewaijun.lee@gmail.com (A.L.); weiping876@hotmail.com (P.W.); wanglu2013@dhu.edu.cn (L.W.); 3 Université Paris Descartes, PRES Sorbonne Paris Cité, CNRS UMR 860, Laboratoire de Chimie et de Biochimie Pharmacologiques et Toxicologique, 45, rue des Saints Peres, 75006 Paris, France; 4 CQM—Centro de Química da Madeira, MMRG, Universidade da Madeira, Campus da Penteada, 9020-105 Funchal, Portugal; 5 Laboratoire de Chimie de Coordination du CNRS, 205 Route de Narbonne, BP 44099, 31077 Toulouse CEDEX 4, France; anne-marie.caminade@lcc-toulouse.fr; 6 Université de Toulouse, UPS, INPT, 31077 Toulouse CEDEX 4, France

**Keywords:** PAMAM dendrimers, β-CD, gold nanoparticles, gene silencing, siRNA

## Abstract

We describe a safe and highly effective non-viral vector system based on β-cyclodextrin (β-CD)-modified dendrimer-entrapped gold nanoparticles (Au DENPs) for improved delivery small interfering RNA (siRNA) to glioblastoma cells. In our approach, we utilized amine-terminated generation 5 poly(amidoamine) dendrimers partially grafted with β-CD as a nanoreactor to entrap Au NPs. The acquired β-CD-modified Au DENPs (Au DENPs-β-CD) were complexed with two different types of therapeutic siRNA (B-cell lymphoma/leukemia-2 (Bcl-2) siRNA and vascular endothelial growth factor (VEGF) siRNA). The siRNA compression ability of the Au DENPs-β-CD was evaluated by various methods. The cytocompatibility of the vector/siRNA polyplexes was assessed by viability assay of cells. The siRNA transfection capability of the formed Au DENPs-β-CD vector was evaluated by flow cytometric assay of the cellular uptake of the polyplexes and Western blot assays of the Bcl-2 and VEGF protein expression. Our data reveals that the formed Au DENPs-β-CD carrier enables efficiently delivery of siRNA to glioma cells, has good cytocompatibility once complexed with the siRNA, and enables enhanced gene silencing to inhibit the expression of Bcl-2 and VEGF proteins. The developed Au DENPs-β-CD vector may be used for efficient siRNA delivery to different biosystems for therapeutic purposes.

## 1. Introduction

Gene therapy is becoming a promising strategy for cancer therapy by transfecting genetic materials (such as DNA [1,2,3], RNA [4,5] and antisense oligonucleotides [6,7]) into target cancer cells to achieve the therapeutic purposes. Among that, RNA interference has been considered as one of the most potential strategies for cancer therapy through silencing the specific genes up-regulated in cancer cells or involved in cell division [8,9]. Small interfering RNA (siRNA) has played a central role for RNA interference. 

For efficient siRNA delivery, one has to consider to use a carrier system owing to the electrostatic repulsion of the negatively-charged cell membrane and the negatively-charged siRNA backbone [10,11]. For gene delivery, non-viral vectors are increasingly employed considering their better bio-safety profile than viral ones, relative ease to be synthesized, targeting specificity to cell/tissue after surface modification [12,13,14], structural diversity, and capacity to transfect plasmid of varying sizes [15,16]. The currently used non-viral vectors such as cationic lipids [17,18], polymers [12,19,20], polypeptides [21,22], and inorganic nanoparticles (NPs) [23,24] can be used to form vector/DNA polyplexes at the nanoscale [10,11,25]. In any case, development of novel vector systems for safe and effective transfection of siRNA still remains a critical research area [26,27,28,29,30,31,32].

Poly(amidoamine) (PAMAM) dendrimers are a class of monodispersed macromolecules [33,34,35] with branched interior and well-fixed molecular conformation and controlled surface functionalities [36,37,38,39]. These properties of PAMAM dendrimers make them act as desired carrier systems for non-viral siRNA delivery [13,40,41,42]. In this direction, several studies have reported that by functional modifications of generation 5 (G5) PAMAM dendrimers via dendrimer periphery modification of polyethylene glycol chains [43] or entrapment of gold (Au) NPs within dendrimer internal cavities [44], the cytotoxicity of the vector has been significantly reduced, while the gene transfection efficiency has been prominently enhanced [14,44,45,46,47]. Previously, we have shown that the Au NP entrapment within G5 PAMAM dendrimers to form dendrimer-entrapped Au NPs (Au DENPs) is beneficial to reduce the cytotoxicity of dendrimers and simultaneously enhance the gene delivery efficiency [44]. On one hand, the entrapped Au NPs are able to compensate the dendrimer terminal amine cytotoxicity by reducing the density of amine groups, thus improving the cytocompatibility of the dendrimers; on the other hand, the Au NPs entrapped helps to maintain the globular conformation of the dendrimer, thereby enhancing the compaction capacity of DNA.

Recently, we have used β-cyclodextrin (β-CD) to modify the surface of G5 PAMAM dendrimers and used the β-CD-modified G5 dendrimers for the synthesis of Au NPs within the dendrimers [48]. The prepared β-CD-modified Au DENPs (Au DENPs-β-CD) displayed weakened cytotoxicity and enhanced DNA compression capability, and enabled the enhanced transfection of plasmid DNA (pDNA) encoding either luciferase gene or enhanced the green fluorescent protein gene into 293T cells. The enhanced gene delivery efficiency could be resulted from the entrapped Au NPs. Meanwhile, the attached β-CD moieties might facilitate the release of pDNA or the siRNA complex from endosomes after endocytic uptake [49]. These promising results strongly stimulate us to further explore the performance of the Au DENPs-β-CD to deliver siRNA to silence genes in cancer cells.

**Scheme 1:**
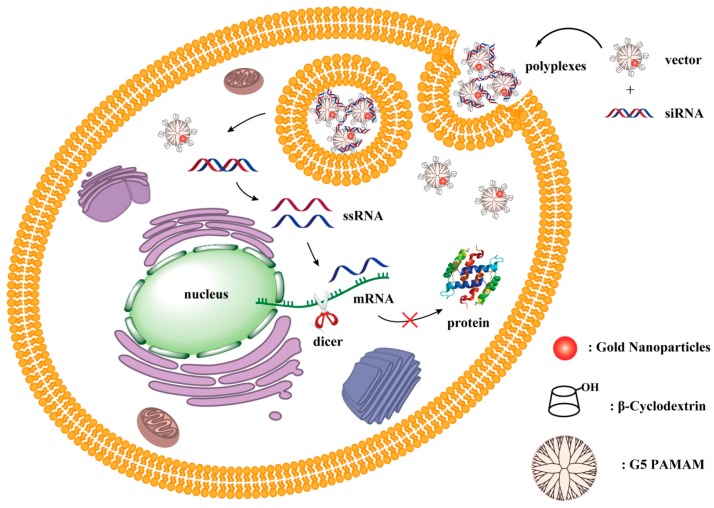
Schematic illustration of the gene delivery process.

In this work, we prepared the Au DENPs-β-CD vector according to the procedure previously reported [48]. The prepared Au DENPs-β-CD were employed as a vector to compact two different types of siRNA including B-cell lymphoma 2 (Bcl-2) siRNA and vascular endothelial growth factor (VEGF) siRNA under the appropriate N/P (dendrimer terminal amine/siRNA phosphate) ratios for oncogene silencing (Scheme 1). The siRNA compaction ability of the vector was explored by gel electrophoresis, dynamic light scattering, and zeta potential measurements. The cytocompatibility of the prepared vector/siRNA polyplexes was also analyzed by cell viability assay. Furthermore, flow cytometry assay and confocal microscopic observations of cells were used to assess the efficiency of gene transfection of the vector/siRNA polyplexes. Finally, we used Western blot assay to prove the oncogene silencing efficiency of the Au DENPs-β-CD/siRNA polyplexes in glioblastoma cancer cells.

## 2. Results and Discussion

### 2.1. Characterization of Vector/siRNA Polyplexes 

**Figure 1:**
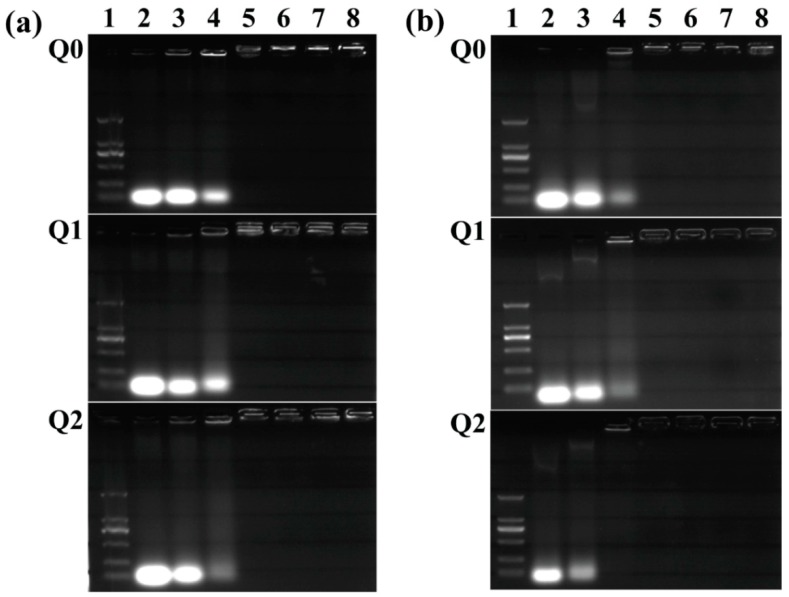
Gel retardation assay of B-cell lymphoma/leukemia-2 (Bcl-2) siRNA (**a**) and vascular endothelial growth factor (VEGF) siRNA (**b**) complexed with Q0, Q1 and Q2 at various N/P ratios, respectively. Lane 1: DNA marker; lane 2: N/P = 0.25:1; lane 3: N/P = 0.5:1; lane 4: N/P = 1:1; lane 5: N/P = 2:1; lane 6: N/P = 3:1; lane 7: N/P = 4:1; and lane 8: N/P = 5:1.

Herein, we aimed to explore the enhanced delivery efficiency of siRNA using the Au DENPs-β-CD (Q2) vector. Au DENPs without β-CD modification (Q1) and pristine G5.NH_2_ dendrimers (Q0) were used for comparison. We first checked the siRNA compaction capability of the vectors. Agarose gel retardation assay was used to analyze the obtained vector/siRNA polyplexes (Figure 1). Apparently, the migration of both siRNAs is able to be blocked at higher N/P ratios. For Bcl-2 siRNA, all vectors fully inhibit the siRNA migration at the N/P ratio of 2:1 or above (Figure 1a). For VEGF siRNA, Q0 and Q1 retard the siRNA migration at the N/P ratio of 2:1 or above (Figure 1b), similar to Bcl-2 siRNA. It seems that Q2 displays a better compaction capacity of VEGF siRNA than Q0 and Q1, and the siRNA migration can be completely retarded at the N/P ratio above 1:1. Clearly, the conjugation of β-CD onto G5 dendrimers is beneficial for enhanced siRNA compaction, in accordance with our previous study [48]. These results demonstrated that both Bcl-2 siRNA and VEGF siRNA could be efficiently compressed by the vectors at N/P ratios greater than 2:1.

**Figure 2:**
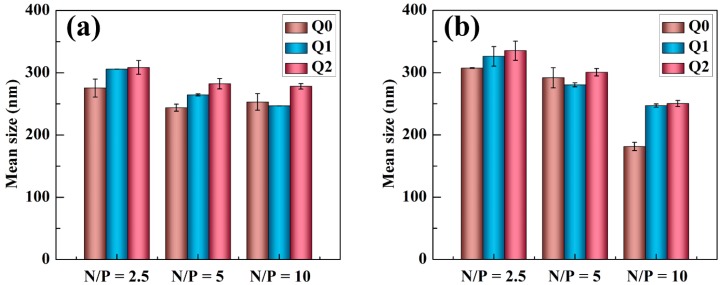
Mean hydrodynamic size of Bcl-2 siRNA (**a**) and VEGF siRNA (**b**) complexed with Q0, Q1, and Q2 at N/P ratios of 2.5:1, 5:1, or 10:1, respectively (mean ± SD, *n* = 3).

DLS and zeta potential measurements were utilized to characterize the vector/siRNA polyplexes (Figure 2 and Table S1, Supporting Information). Apparently, the hydrodynamic particle size of the vector/siRNA polyplexes decreases with the N/P ratio. This may be attributed to the situation that at a higher N/P ratio, more vector materials are present and the siRNA can be more compacted to have a smaller size. It appears that polyplexes composed of the vectors with Au NPs entrapped have a slightly larger size than those gained using dendrimers without the entrapment of Au NPs, which is different from the vector/pDNA in our earlier work [44]. This difference is probably owing to the much smaller molecular weight of siRNA, and the size of the polyplexes could be mainly derived from that of the vectors.

**Figure 3:**
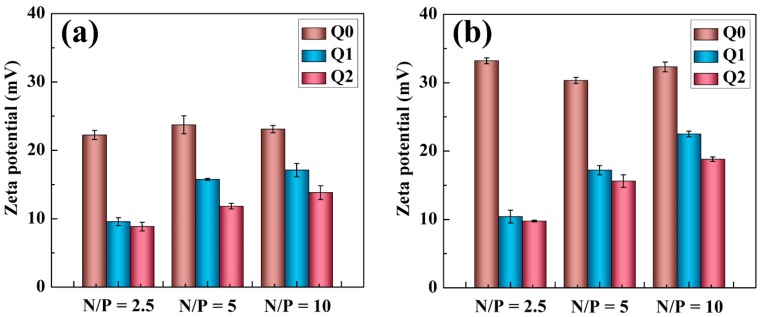
Surface potentials of Bcl-2 siRNA (**a**) and VEGF siRNA (**b**) complexed with Q0, Q1, and Q2 at N/P ratios of 2.5:1, 5:1 or 10:1, respectively (mean ± SD, *n* = 3).

We determined the surface potentials of the vector/siRNA polyplexes under the given N/P ratios (Figure 3). Clearly, at the same N/P ratios, the surface potential of the polyplexes obeys the order of Q0/siRNA > Q1/siRNA > Q2/siRNA for both Bcl-2 siRNA (Figure 3a) and VEGF siRNA (Figure 3b). These data suggest that the modification of β-CD on the dendrimer periphery and the entrapment of Au NPs within the dendrimer internal cavity reduce the density of dendrimer surface amines, thus decreasing the surface potentials of the formed polyplexes. This is beneficial for them to have an improved cytocompatibility (see below). However, the surface potentials of the Q1/siRNA and Q2/siRNA are still positively charged. Overall, the positive surface potential along with the appropriate hydrodynamic sizes of the polyplexes may allow them to be used for effective gene delivery [48].

### 2.2. Cytotoxicity Assay

**Figure 4:**
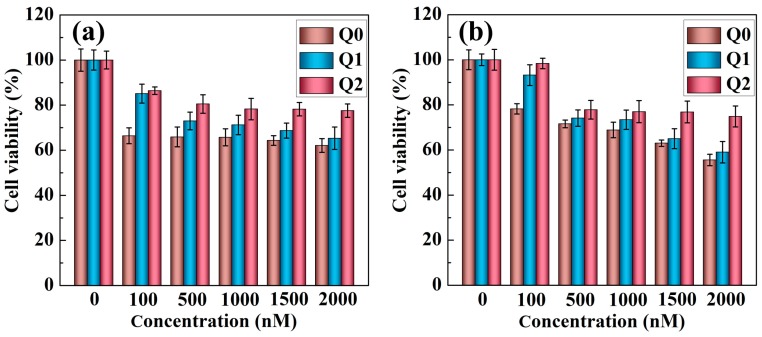
MTT viability assay of Uppsala 87 Malignant Glioma (U87MG) cells treated with the vector/Bcl-2 siRNA (**a**) and vector/VEGF siRNA; and (**b**) polyplexes under distinct vector concentrations (mean ± SD, *n* = 6).

MTT assay of cell viability was next carried out to test the cytotoxicity of vector/siRNA polyplexes (Figure 4). The cell viability is reduced with the vector concentration. The cytotoxicity of the vector/siRNA polyplexes obeys the order of Q2/siRNA < Q1/siRNA < Q0/siRNA at the same vector concentration for either Bcl2 siRNA (Figure 4a) or VEGF siRNA (Figure 4b). Even at the highest vector concentration of 2000 nM, the cells incubated with the Q2/siRNA polyplexes still display a viability higher than 80%, while the cells co-cultured with the Q0/siRNA polyplexes show a viability lower than 60%. Our results suggest that Q2 could be adopted as a relatively ‘safe’ gene delivery vector when compared with Q0 and Q1. Our data emphasize that the dendrimer periphery modification with β-CD and the internal loading of Au NPs are beneficial to compromise the cytotoxicity of G5 PAMAM dendrimers.

### 2.3. Uptake of Vector/siRNA Polyplexes by Cancer Cells 

**Figure 5:**
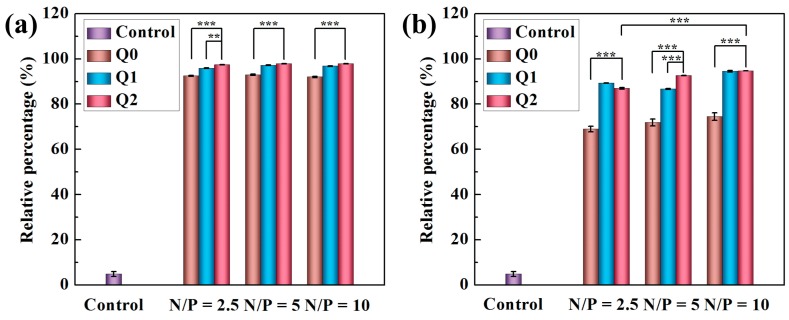
Flow cytometry analysis of U87MG cells transfected using vector/Cy3-Bcl-2 siRNA (**a**) and vector/Cy3-VEGF siRNA (**b**) polyplexes at the N/P ratios of 2.5:1, 5:1, and 10:1, respectively. Cells without treatment were used as control. Data are shown with mean ± SD (*n* = 3). For (**a**,**b**), ** and *** mean that the *p* values of statistical analyses are <0.01 and <0.001, respectively.

The cellular uptake of the vector/siRNA polyplexes was investigated by flow cytometry analysis (Figure S1, Supporting Information). As shown in Figure 5, for both vector/Cy3-Bcl-2 siRNA and vector/Cy3-VEGF siRNA polyplexes, the percentages of fluorescent cells associated with the Cy3-labeled siRNA are relatively higher at the higher N/P ratios of the polyplexes. Under the same conditions, it appears that Bcl-2 siRNA (Figure 5a) can be more significantly uptaken by the U87MG cells than VEGF siRNA (Figure 5b). The percentage of cells having the Cy3 fluorescence signals after transfection by the vector/Cy3-Bcl-2 siRNA polyplexes is higher than 80% for all different vectors. The ability of vector to deliver Bcl-2 siRNA and VEGF siRNA follows the tendency of Q0 < Q1 < Q2 at N/P ratios of 5 and 10. It should be noted that Q2 possesses a lower surface potential, thus being more liable to show enhanced transfection efficiency of genes at high N/P ratios.

**Figure 6:**
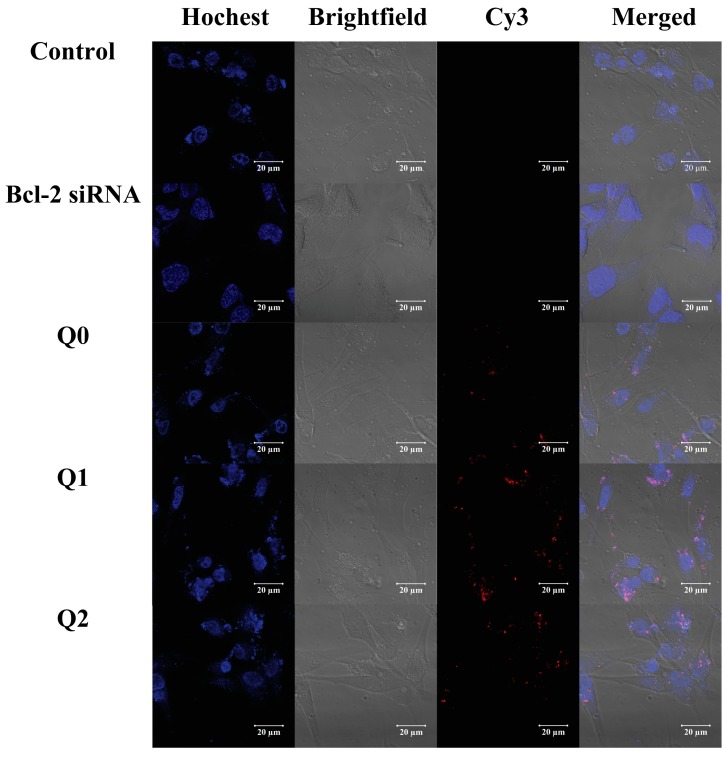
Confocal microscopic images (63×) of U87MG cells showing the intracellular localization of the vectors/Cy3-Bcl-2 siRNA polyplexes that were prepared with an N/P of 5:1 (red: Cy3-labeled siRNA; blue: Hoechst 33342 stained cell nuclei).

Confocal microscopic imaging was used to further study the transcellular pathways and localization of the vector/siRNA polyplexes at the N/P ratio of 5:1. As illustrated in Figure 6, control cells without treatment just display the Hoechst 33342-stained cell nuclei, which is blue fluorescent. The cells incubated with free Cy3-Bcl-2 siRNA without vector just displays the blue fluorescence associated to the Hoechst 33342 staining, similar to the control cells. This indicates that free Cy3-Bcl-2 siRNA is not able to be transfected into the cells due to its intrinsic negative charge. In contrast, Cy3-Bcl-2 siRNA complexed with all three different vectors can be transfected within the cancer cells, as red fluorescence signals in all cases can be seen in the cytoplasm of cells. The intensity of the red fluorescence signals of cells follows the order of Q0 < Q1 < Q2 under the same condition, suggesting that the siRNA delivery efficiency follows the same order, in agreement with the flow cytometry assay data. For the Cy3-VEGF siRNA delivery (Figure S2, Supporting Information), the same trend of cellular uptake of the polyplexes can be seen.

### 2.4. Gene Silencing with VEGF and Bcl-2 siRNAs

**Figure 7:**
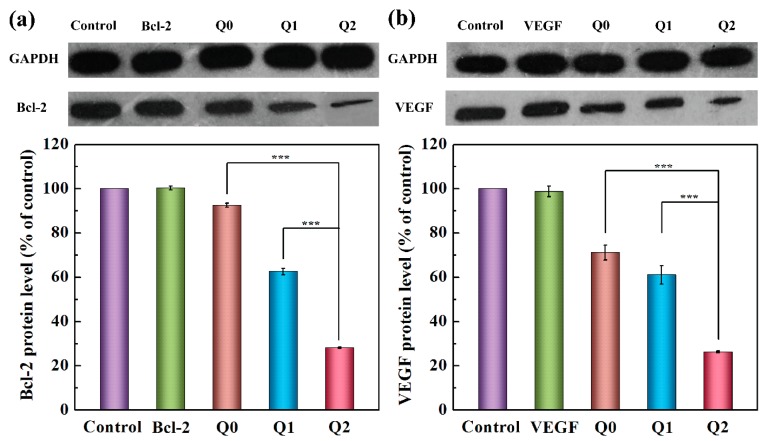
Relative Bcl-2 (**a**) and VEGF (**b**) protein expression levels in U87MG cells transfected with vector/siRNA polyplexes at an N/P ratio of 5:1. The GAPDH protein was used as a reference for the Western blot data. For (**a**,**b**), *** means that the *p* values of statistical analyses are <0.001.

With the goal to study the effective siRNA delivery using the designed vector systems, Western blot analysis was undertaken to explore the gene silencing capabilities of vector/siRNA polyplexes at the N/P ratio of 5:1. We used naked siRNA without any protection as a control and GAPDH protein was adopted as the reference protein. As shown in Figure 7a, the Bcl-2 gene is not able to be silenced by naked Bcl-2 siRNA, which is similar to the control cells. In contrast, decreased expression of Bcl-2 protein can be seen in cells treated with all vector/siRNA polyplexes, and the gene silencing efficacy follows the order of Q2 > Q1 > Q0. Similarly, VEGF siRNA is also able to be transfected after complexation with the vectors of Q0, Q1, and Q2 to lower the expression of VEGF protein (Figure 7b), and the gene silencing trend follows the same order. These results prove that the Au DENPs-β-CD (Q2) possess excellent siRNA transfection ability and the formed polyplexes are able to silence the genes in cancer cells for effective gene therapy applications.

## 3. Experimental Section

### 3.1. Synthesis of Au DENPs-β-CD

G5.NH_2_, Au DENPs, and Au DENPs-β-CD were obtained using the same procedures described in our previous work [48]. For clarity, the structure of G5.NH_2_ PAMAM dendrimer and β-CD can be seen in Figure S3 (Supporting Information). Besides, for simplicity, the above materials were referred to as Q0, Q1, and Q2, respectively.

### 3.2. Preparation of Vectors/siRNA Polyplexes

Bcl-2 and VEGF siRNAs were respectively complexed with vectors with various N/P ratios. Both vectors and siRNA (1 μg) were dissolved in phosphate buffered saline (PBS, 0.01 mol/L, pH 7.4) to reach an ultimate volume of 100 μL, then blended gently and incubated for 20 min at room temperature.

### 3.3. Gene Silencing with VEGF and Bcl-2 siRNAs 

U87MG cells were incubated into 12-well flat-bottomed plates at a density of 1 × 10^5^ cells/well as described above. When the cells were brought to a 60–70% confluence, the medium in each well was substituted with 1 mL of fresh DMEM containing 100 μL of vector/siRNA polyplexes prepared using 1 μg of siRNA at the N/P ratio of 5:1. After incubation for 4 h, the cell medium was exchanged to fresh DMEM supplemented with 10% FBS and the cells were then cultivated for additional 48 h. Then, the cells were treated with pre-chilled PBS and digested with lysis buffer (400 μL/well) for 30 min under ice bath. The lysates were centrifuged at 12,000 rpm for 5 min at 4 °C. Thereafter, the supernatants were collected for Western-blot assay according to protocols in our previous work [13]. Both the Bcl-2 and VEGF proteins were detected using a Western Lightning^®^ Plus-ECL (Enhanced Chemiluminescence) Kit (PerkinElmer, Inc., Boston, MA, USA). More detailed information of experimental can be seen in Supporting Information.

## 4. Conclusions

To summarize, we used the synthesized Au DENPs-β-CD as a carrier for highly efficient delivery of siRNA to glioblastoma cells. The Au DENPs-β-CD are much more efficient to compact siRNA under appropriate N/P ratios, and exhibit lower cytotoxicity and better siRNA delivery efficiency than the pristine G5.NH_2_ dendrimers and the Au DENPs without β-CD modification. These results are related to the dendrimer surface modification of β-CD and dendrimer internal cavity entrapment of Au NPs. In addition, with the assistance of the Au DENPs-β-CD vector, both Bcl-2 and VEGF siRNAs are able to be transfected to silence of glioblastoma cancer cells by downregulation of the respective proteins. Strongly, our study suggests that Au DENPs-β-CD may be employed as a prospective carrier for safe and effective transfection of siRNA to treat a variety of cancer cells based on specific siRNAs.

## Supplementary Materials

The following are available online at http://www.mdpi.com/2079-4991/8/3/131/s1. Figure S1: Flow cytometry analysis spectra; Figure S2: Confocal microscopic images; Figure S3: Molecular structures of G5.NH_2_ PAMAM dendrimers and β-CD; Table S1: Polydispersity index (PDI) of siRNA complexed with vectors.
