# Supplementary material for: Enhanced Delivery of Therapeutic siRNA into Glioblastoma Cells Using Dendrimer-Entrapped Gold Nanoparticles Conjugated with β-Cyclodextrin
Source: Nanomaterials (Basel). 2018 Feb 27;8(3):131. doi: 10.3390/nano8030131 (PMC5869622; doi:10.3390/nano8030131)
Supplement: Supplementary file 1 [file nanomaterials-08-00131-s001.pdf]

nanomaterials

MDPI

Supplementary Information

Enhanced Delivery of Therapeutic siRNA into Glioblastoma Cells Using Dendrimer-Entrapped Gold Nanoparticles Conjugated with β-Cyclodextrin

1. Part of Experimental Section

1.1. Materials

G5 PAMAM dendrimers were purchased from Dendritech (Midland, MI, USA). -CD and N, N'-carbonyl diimidazole (CDI) were purchased from J and K Chemical (Shanghai, China). Dimethylsulfoxide (DMSO) was from Lingfeng (Shanghai, China). Agarose was from Biowest (Nuaillé, France). MTT was from Sangon (Shanghai, China). U87MG cells (a human primary glioblastoma cell line) were obtained from Institute of Biochemistry and Cell Biology (the Chinese Academy of Sciences, Shanghai, China). Dulbecco modified Eagle's medium (DMEM) and fetal bovine serum (FBS) were purchased from Gibco (Carlsbad, CA, USA). Penicillin and streptomycin were from Gino (Shanghai, China). Bcl-2 siRNA (sense, 5′-GUA CAU CCA UUA UAA GCU G dtdt-3′; antisense, 5′-CAG CUU AUA AUG GAU GUA C dtdt-3′), VEGF siRNA (sense, 5′-GGA GUA CCC UGA UGA GAU C dtdt-3′; antisense, 5′-GAU CUC AUC AGG GUA CUC C dtdt-3′), Cy3-labeled Bcl-2 siRNA and Cy3-labeled VEGF siRNA were obtained from Shanghai GenePharma Co., Ltd. (Shanghai, China). Monoclonal antibodies, including anti-Bcl-2, anti-VEGF, and the IgG isotype control antibody were obtained from Maibio (Shanghai, China). GAPDH siRNA was purchased from Merck Millipore (Shanghai, China). PBS buffer (pH 7.4) was provided from Sinopharm Chemical Reagent Co., Ltd. (Shanghai, China). All materials or chemicals were used as received.

1.2. Agarose Gel Retardation Assay

The agarose gel retardation assay was used to determine the compaction ability of vectors with siRNA. Agarose gel (1.0% *w*/*v*) containing ethidium bromide (0.5 g/mL) was prepared with tris-borate-ethylenediaminetetraacetic acid buffer. The vector/siRNA polyplexes were prepared using 1 g of siRNA under various N/P ratios ranging from 0.25:1 to 5:1. The final volume of the formed polyplexes was 20 μL. Gel electrophoresis was carried out for 45 min at 80 V. The band signals were analyzed through a gel image analysis system (Shanghai FURI Science and Technology, Shanghai, China) at 312 nm.

1.3. Dynamic Light Scattering (DLS) and Zeta Potential Measurements

Different vector/siRNA polyplexes were prepared using 5 μg of siRNA at the N/P ratios of 2.5:1, 5:1, or 10:1. After diluted to a final volume of 1 mL using PBS, the polyplexes were subjected to DLS and zeta potential measurements using a Malvern Zetasizer Nano ZS system (Malvern Instruments, Worcestershire, UK) equipped with a standard 633 nm laser.

1.4. Cytotoxicity Assay

MTT assay was used to evaluate the cytotoxicity of vector/siRNA polyplexes. In brief, U87MG cells were cultivated in 96-well plates at a density of 1×10^4 cells/well with 200 L of DMEM containing 10% FBS, 100 U/mL penicillin, and 100 g/mL streptomycin at 37 °C and 5% CO_2 for 24 h. Then, the medium of each well was replaced with 200 L of fresh DMEM containing 20 L of vector/siRNA polyplexes prepared using 1 μg of siRNA with vectors at a concentration ranging from 100 to 2000 nM. PBS was used as the control. After incubation for 24 h, MTT (5.0 mg/mL, 20 L/well) was added to each well and the cells were cultured for additional 4 h. Subsequently, the formazan crystals

in each well were dissolved by 150 L of DMSO. The absorbance was measured by a Thermo Scientific Multiskan MK3 ELISA reader (Thermo Scientific, Waltham, MA, USA) at 570 nm. Mean and standard deviation of six parallel wells was reported for each sample.

1.5. Flow Cytometry

Flow cytometry (BD FACS Calibur, Franklin Lakes, NJ, USA) was used to check the cellular uptake of the vector/siRNA polyplexes. U87MG cells were prepared in 24-well plates at a density of 1×10^5 cells per well in 1 mL of DMEM, then the cells were incubated overnight to bring the cells to confluence (over 70%). Afterwards, the medium in each well was replaced with 1 mL of fresh DMEM containing 100 L of vector/Cy3-siRNA polyplexes prepared using 1 μg of Cy3-siRNA at the N/P ratios of 2.5:1, 5:1, and 10:1, respectively. Simultaneously, non-treated cells were utilized as the control. After incubation for 4 h, the cells were washed three times with PBS, trypsinated, centrifuged, and resuspended in 1 mL of PBS before flow cytometry analysis. The cells were gated (G1) on a forward scatter/side scatter (FSC-H/SSC-H) dot plot and the fluorescence was measured in the FL2-H channel. For each vector with different N/P ratios, the experiment was repeated three times and 1×10^4 cells were counted every time.

1.6. Confocal Microscopy

The intracellular localization of vector/Cy3-siRNA polyplexes was observed by laser scanning confocal microscope. U87MG cells were cultured into 24-well plates at a density of 1×10^5 cells/well for 24 h as described above, then transfected by the vector/Cy3-siRNA polyplexes with an N/P ratio of 5:1 according to the same process used for flow cytometry assay. After 4 h incubation, the cells were washed three times with PBS and fixed with 2.5% glutaraldehyde (300 L/well) for 15 min at 4 °C. Then the cells were washed 3 times with PBS again and counterstained with Hoescht 33342 (1 g/mL for each well) for 30 min at 37 °C to stain the cell nuclei. Images were acquired using a Zeiss confocal microscopy (Jena, Germany) with a 63× oil immersion lens.

1.7. Statistical Analysis

One-way ANOVA statistical analysis was performed to evaluate the experimental data. A p value of 0.05 was selected as the significance level, and the data were indicated with (*) for $p < 0.05$, (**) for $p < 0.01$, and (***) for $p < 0.001$, respectively.

2. Figures and Tables

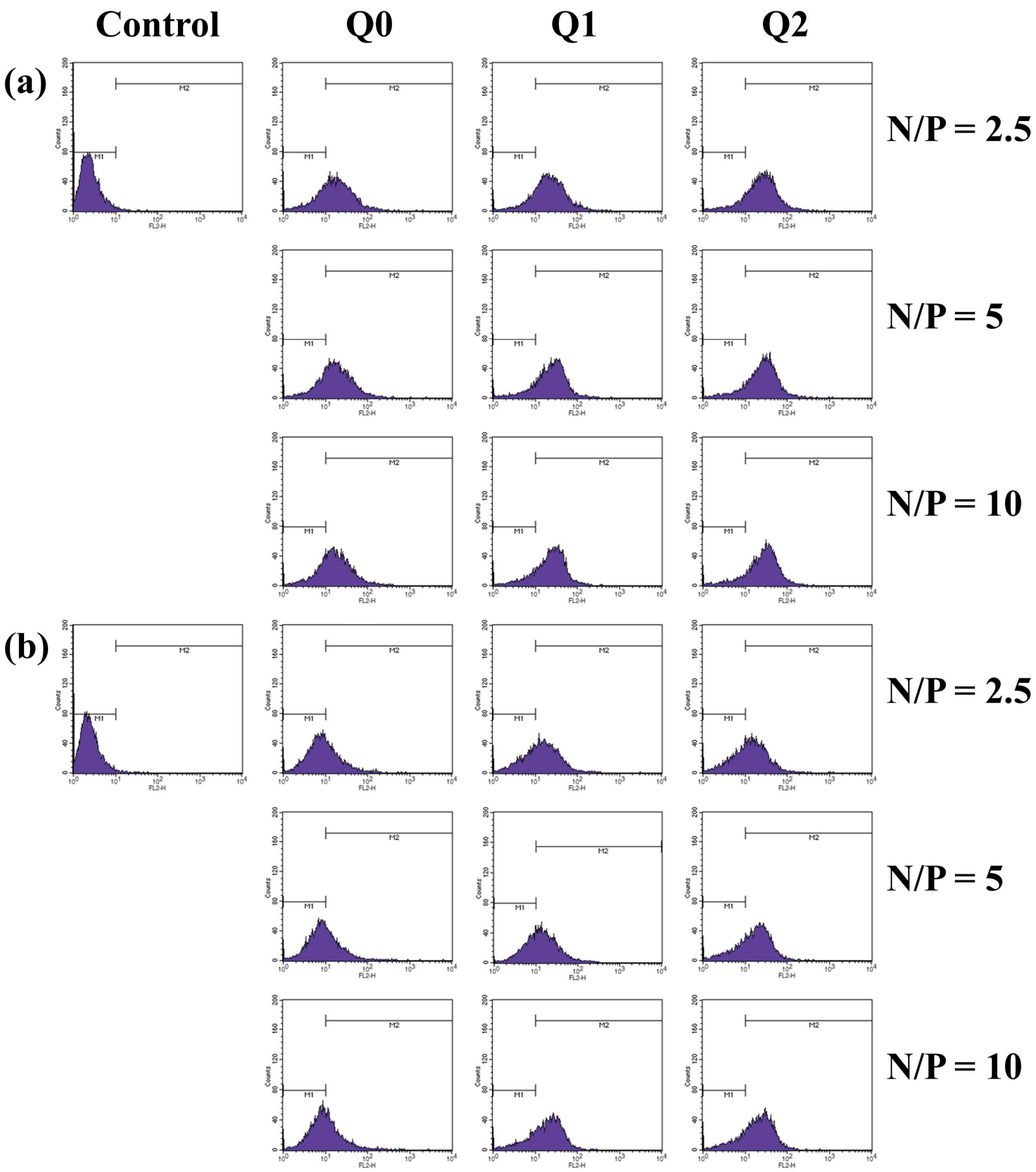

Figure S1. Flow cytometry analysis of U87MG cells transfected using vector/Cy3-Bcl-2 siRNA (**a**) and vector/Cy3-VEGF siRNA; and (**b**) polyplexes at the N/P ratios of 2.5:1, 5:1, and 10:1, respectively. Cells without treatment were used as control.

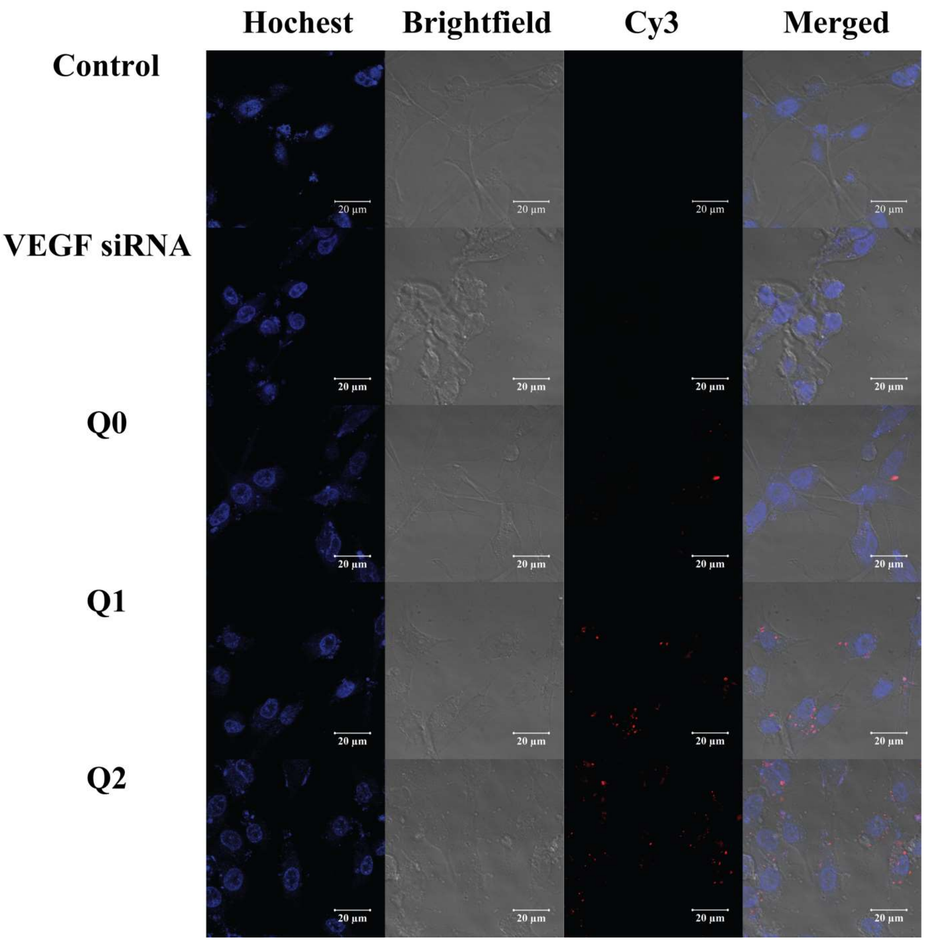

Figure S2. Confocal microscopic images (63×) of U87MG cells showing the intracellular localization of the vectors/Cy3-VEGF siRNA polyplexes that were prepared with an N/P of 5:1 (red: Cy3-labeled siRNA; blue: Hoechst 33342 stained cell nucleus nuclei).

G5 PAMAM:

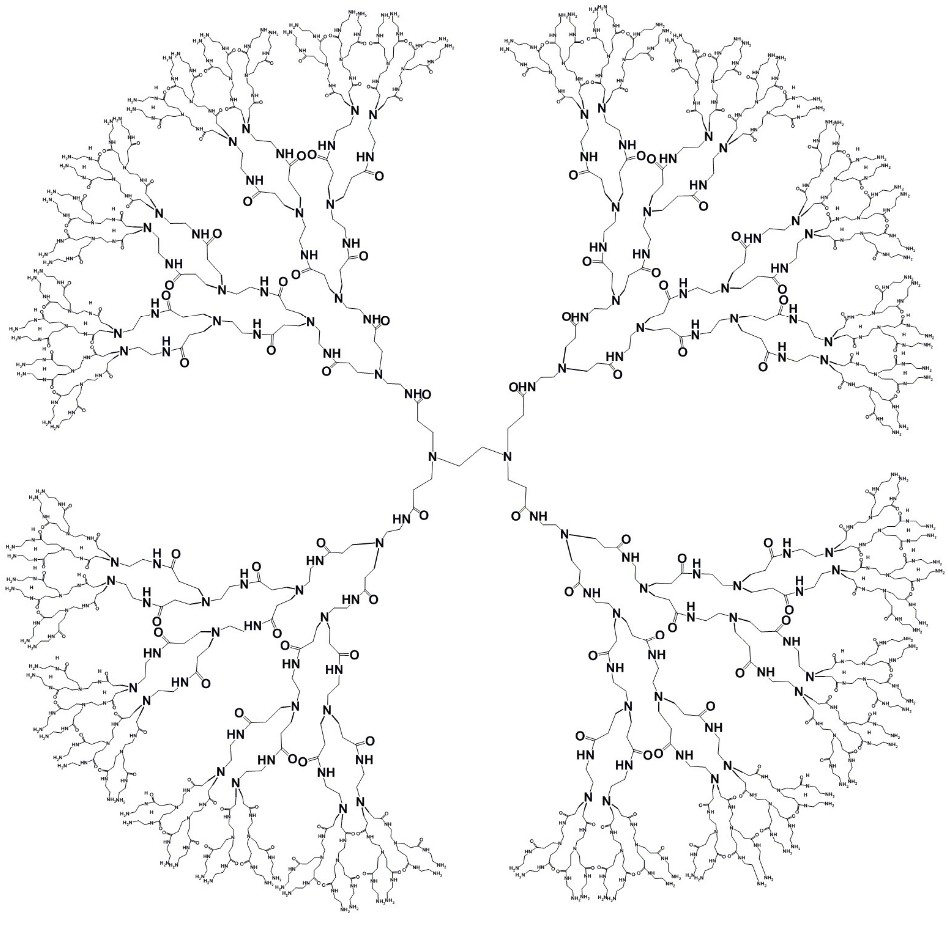

β-Cyclodextrin:

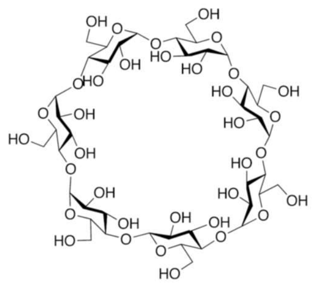

Figure S3. Molecular structures of G5.NH_2 PAMAM dendrimers and β-CD.

Table S1. Polydispersity index (PDI) of Bcl-2 siRNA and VEGF siRNA complexed with Q0, Q1, and Q2 at N/P ratios of 2.5:1, 5:1, or 10:1, respectively (mean ± SD, n = 3).

Vector/siRNA polyplexes	Q0/Bcl-2	Q1/Bcl-2	Q2/Bcl-2	Q0/VEGF	Q1/VEGF	Q2/VEGF
N/P = 2.5	0.11 ± 0.01	0.36 ± 0.09	0.34 ± 0.03	0.15 ± 0.05	0.32 ± 0.02	0.27 ± 0.02
N/P = 5	0.15 ± 0.01	0.34 ± 0.10	0.40 ± 0.02	0.15 ± 0.02	0.40 ± 0.09	0.34 ± 0.03
N/P = 10	0.14 ± 0.02	0.49 ± 0.07	0.37 ± 0.02	0.13 ± 0.02	0.37 ± 0.05	0.42 ± 0.06

© 2018 by the authors. Submitted for possible open access publication under the terms and conditions of the Creative Commons Attribution (CC BY) license (http://creativecommons.org/licenses/by/4.0/).
